# A mixed methods multiple case study to evaluate the implementation of a care pathway for colorectal cancer surgery using extended normalization process theory

**DOI:** 10.1186/s12913-020-06011-w

**Published:** 2021-01-04

**Authors:** R. van Zelm, E. Coeckelberghs, W. Sermeus, A. Wolthuis, L. Bruyneel, M. Panella, K. Vanhaecht

**Affiliations:** 1 Leuven Institute for Healthcare Policy, Katholieke Universiteit Leuven, Leuven, Belgium; 2 Depertment of Abdominal Surgery, University Hospital Leuven, Leuven, Belgium; 3 Department of Translational Medicine, University of Eastern Piemonte (UPO), Novarra, Italy; 4 Department of Quality, Academic Policy Advisor, University Hospital Leuven, Leuven, Belgium

**Keywords:** Process evaluation, Care pathway, Enhanced recovery, Mixed methods case study, Implementation, Extended normalization process theory (eNPT)

## Abstract

**Background:**

Specific factors that facilitate or prevent the implementation of enhanced recovery protocols for colorectal cancer surgery have been described in previous qualitative studies. This study aims to perform a concurrent qualitative and quantitative evaluation of factors associated with successful implementation of a care pathway (CP) for patients undergoing surgery for colorectal cancer.

**Methods:**

This comparative mixed methods multiple case study was based on a sample of 10 hospitals in 4 European countries that implemented a specific CP and performed pre- and post-implementation measurements. In-depth post-implementation interviews were conducted with healthcare professionals who were directly involved. Primary outcomes included protocol adherence and improvement rate. Secondary outcomes included length of stay (LOS) and self-rated protocol adherence. The hospitals were ranked based on these quantitative findings, and those with the highest and lowest scores were included in this study. Qualitative data were summarized on a per-case basis using extended Normalization Process Theory (eNPT) as theoretical framework. The data were then combined and analyzed using joint display methodology.

**Results:**

Data from 381 patients and 30 healthcare professionals were included. Mean protocol adherence rate increased from 56 to 62% and mean LOS decreased by 2.1 days. Both measures varied greatly between hospitals. The two highest-ranking hospitals and the three lowest-ranking hospitals were included as cases. Factors which could explain the differences in pre- and post-implementation performance included the degree to which the CP was integrated into daily practice, the level of experience and support for CP methodology provided to the improvement team, the intrinsic motivation of the team, shared goals and the degree of management support, alignment of CP development and hospital strategy, and participation of relevant disciplines, most notably, physicians.

**Conclusions:**

Overall improvement was achieved but was highly variable among the 5 hospitals evaluated. Specific factors involved in the implementation process that may be contributing to these differences were conceptualized using eNPT. Multidisciplinary teams intending to implement a CP should invest in shared goals and teamwork and focus on integration of the CP into daily processes. Support from hospital management directed specifically at quality improvement including audit may likewise facilitate the implementation process.

**Trial registration:**

NCT02965794.

US National Library of Medicine, ClinicalTrials.gov. Registered 4 August 2014.

**Supplementary Information:**

The online version contains supplementary material available at 10.1186/s12913-020-06011-w.

## Background

Over the past 15 years, procedures for colorectal cancer surgery have been standardized with the introduction of enhanced recovery protocols (ERPs), which are also known as enhanced recovery after surgery (ERAS) protocols [1]. The fourth update of the internationally-recognized ERAS protocol for this indication was published in 2018 [2]. Efficacy and safety of these protocols have been studied extensively, leading to the are feasible, safe, and result in improved postoperative outcomes [3]. However, adherence to the interventions recommended by the ERPs seems to be challenging. Although several groups have already presented evidence suggesting a direct relationship between adherence rates (ARs) and patient outcomes [4–7], reported ARs vary greatly.

Several groups have explored the use of ERPs and have attempted to identify relevant processes, facilitators, and barriers to their implementation. Gotlib Conn et al. (2015) and Gramlich et al. (2017) suggested that the implementation of ERPs involves complex cognitive and social processes. Notably, the participation of an individual serving as a “local champion” and relationship-building capacity are perceived as important factors involved in the implementation of these protocols [8, 9]. Other studies, including a systematic review of 53 studies that focused on the implementation of ERPs in multiple surgical specialties, identified adaptation of a given ERP to local circumstances as a critical facilitator, including its alignment with evidence-based practice, leadership, teamwork, staff education, monitoring, and feedback. Barriers to implementation included resistance to change, lack of stakeholder buy-in, lack of resources, and rotating residents [10–12].

Qualitative research approaches have provided detailed insight into the implementation process and have identified facilitators and barriers in routine clinical practice. In this study, we perform a combined quantitative and qualitative evaluation to generate comprehensive insight into the factors that promote and prevent the implementation of ERPs. This study is the final part of a series of connected studies [13–15] that together provide a process evaluation of pathways associated with colorectal cancer surgery.

Care pathways (CPs) have been introduced as a strategy to improve adherence to recommended care [16, 17]. CPs are complex interventions that structure care around individual patient needs, combine evidence based key interventions, feedback on the current care process and strategies for improvement [18]. As reported in our previous publications, the hospitals participating in this evaluation received feedback on their care process via feedback meetings and a feedback report. As a next step, a model CP based on the ERAS protocol was delivered to all teams and was explained in an on-site quality improvement workshop. Subsequently, participating teams implemented the model CP or adapted their existing local CP. This intervention is described in detail in the study protocol [19]. An earlier, qualitative study was performed to explore this implementation process [15], and a quantitative effect study [14] generated several implications for further research. These implications are addressed in this study.

Our goal is to evaluate the implementation of a CP for colorectal cancer surgery in 10 European hospitals. A multiple case study design was used to interpret and to explain relationships between quantitative data, which focused on the improvement of protocol adherence and reduced lengths of stay (LOSs), and qualitative findings, which included the perspectives of the participating healthcare professionals. We anticipate that an analysis of combined quantitative and qualitative data will enhance our understanding of the implementation process. We are specifically interested in determining how the perspectives of healthcare professionals regarding the CP implementation process in different contexts correlate with the effects and success of its implementation. The research questions include:

1. Which factors explain the difference between pre- and post-implementation performance (LOS and protocol adherence) and thus can be used to promote its improvement?
2. What is the relationship between intended and measured ARs?

## Methods

### Study design and setting

**Fig. 1:**
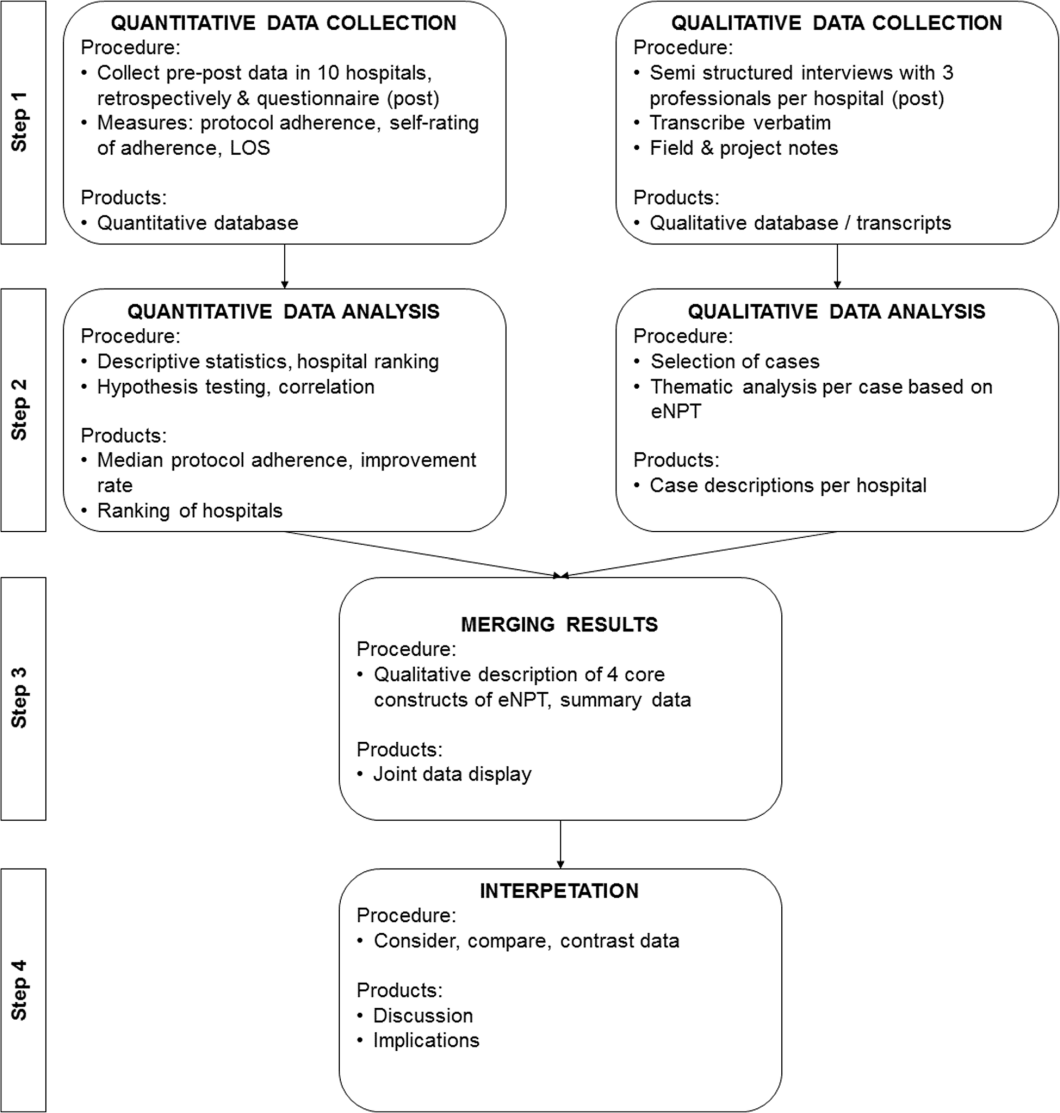
Design of the comparative mixed methods case study (Based on Creswell et al., 2017)

This international mixed methods study was performed in a selected sample of 10 hospitals in Belgium, Germany, France, and the Netherlands. A comparative multiple case study design was used [20]. Fig. 1 presents a diagram of the study design.

### Data collection and measures

#### Quantitative evaluation

For step 1, pre- and post-implementation data were collected from patient records. A sample of 20 consecutive patients from each hospital was included retrospectively for evaluation of pre-pathway implementation (2014); another 20 patients were included for post-pathway implementation (December 2016) to study the impact of the CP on patient and implementation outcomes. Adult patients (≥18 years of age) undergoing elective colorectal cancer surgery (open/laparoscopic) were included. Patients diagnosed with severe dementia (Diagnostic and Statistical Manual of Mental Disorders [DSM] IV), major neurocognitive disorder (DSM V), or severe concomitant disease that might have an impact on short-term outcomes (e.g., life expectancy less than 3 months) were excluded because these patients were unlikely to be able to follow the CP. The local study coordinator was instructed to collect data retrospectively from the patient record using a standardized data extraction form [14].

For step 2, we hypothesized that hospitals that scored lower on pre-implementation ARs would achieve higher improvement scores. Primary outcome measures included median protocol adherence (hospital median of the proportions of relevant interventions as defined by the protocol that were received by each patient) and improvement rates (IRs, i.e., the difference between pre- and post-test ARs). Differences in IRs were analyzed using Mann-Whitney U-tests.

Secondary outcome measures included mean LOS and self-rated protocol adherence (SrA). To determine SrA, additional quantitative data were captured post-implementation with a questionnaire that featured a five-point anchored scale. Each hospital received one questionnaire that assessed the level of intended implementation (0–100%) of each intervention described in the model CP. SrA was determined based on these findings. We hypothesized that there would be positive correlations between SrA and post-test AR and that teams that were actively engaged with the CP with the intent to improve adherence would score higher on assessments of post-test adherence. The relationship between variables was quantified using Pearson’s R.

We generated hospital rankings based on both absolute values and differences in median protocol adherence and mean LOS. The hospital with the highest adherence ranked first and was scored with 1 point; the hospital with the lowest adherence ranked tenth (and received 10 points). Likewise, the hospital with the most substantial improvement in protocol adherence ranked first (1 point), and the hospital that improved least ranked tenth (10 points). The same method was used to score LOS. This resulted in four rankings for each hospital. The total points scored by each hospital contributed to the overall score, with a potential range of 4–40 points.

#### Qualitative evaluation

Step 1 focused on the collection of post-implementation data with in-depth interviews with 3 professionals per hospital. The interviews were based on a semi-structured interview guide and focused on the key elements of process evaluation [21]. A second researcher took field notes, captured non-verbal reactions, and provided reflection during the debriefing that was carried out after each interview. Finally, project notes from feedback and improvement sessions recorded during the project were used to complete the qualitative dataset, which resulted in a “thick description” of the intervention, context, implementation, mechanisms, and perceived outcomes. The methods for the interviews and questionnaires are described in detail in the study protocol [19]. The full topic guide is described in a previous study [15].

In step 2, we set criteria for the selection of cases based on the quantitative data. Since our research is focused on improvement, we included hospitals with the highest (≤10 points) and lowest (≥ 30 points) rankings based on the quantitative data collected as described above. With this method, we would be able to cover the entire spectrum of improvement with a focus on the two extremes. The cases selected were carefully reviewed using extended Normalization Process Theory (eNPT) as a framework. We chose eNPT because it defines, explains, and links key elements that facilitate or impede normalization (defined here as turning a new practice into one that is routine) of complex interventions in a social system [22, 23]. A systematic review by May et al. (2018) focused on the use of NPT as part of the evaluation of a wide range of practices and complex interventions indicated that this framework provided a combination of the conceptual tools needed for the comprehension of implementation as a process [23]. Four core constructs were defined in the third update of eNPT, including two that were focused on context and two addressing the concept of agency, or “the ability to make things happen” (May 2013, p.1). Each core construct is operationalized based on its underlying components. The theory provides four propositions that can be used to explain the normalization of a complex intervention (Table 1) [22]. The original interview guide was based on several theoretical frameworks, including eNPT. As such, all components of eNPT were covered in the interviews.

**Table 1:** Main constructs of eNPT and its 4 propositions (May, 2013)

Core construct	Components	Propositions
**Capability** Possibilities presented by the complex intervention	Workability Integration	The capability of agents to operationalize a complex intervention depends on its workability and integration within a social system.
**Capacity** Social-structural resources available to agents	Material resources Social roles Social norms Cognitive resources	The incorporation of a complex intervention within a social system depends on agents’ capacity to cooperate and coordinate their actions.
**Potential** Social-cognitive resources available to agents	Individual intentions Collective commitment	The translation of capacity into collective action depends on agents’ potential to enact the complex intervention.
**Contribution** What agents do to implement a complex interventions	Coherence Cognitive participation Collective action Reflexive monitoring	The implementation of a complex intervention depends on agents’ continuous contributions that carry forward in time and space.

Qualitative analysis was performed at the hospital level. As such, we combined data, field notes, and reflections on the interviews from different respondents at the same hospital. Cases are summarized in the descriptions included in Additional file 1. The case descriptions were discussed and reviewed by the research team to ensure that they reflected the findings from the original data.

### Data merging and analysis (steps 3 and 4)

The cases were analyzed using “side-by-side” joint displays, which are tables that provide a visual display of combined quantitative and qualitative data [20, 24]. The cases are presented in four displays, one for each core construct of eNPT. Each row represents a case and columns represent quantitative outcome data and healthcare professional experience. This method enabled us to determine if and why any of the individual cases differed with respect to outcomes and experience for each of the eNPT constructs and facilitated the search for patterns and explanations.

## Results

We collected data from 10 independent hospitals including 381 patients and 30 healthcare professionals. The characteristics of each hospital, including the number of beds, the number of colorectal procedures performed each year, the number of patients included from each location, and the backgrounds of each of the healthcare professionals interviewed for this study are shown in Table 2.

**Table 2:** Hospital characteristics and number of patients and interviewees included

Hospital	A	B	C	D	E	F	G	H	I	J	Total
Beds total (dedicated)	200 (0)	1054 (15)	991 (39)	161 (10)	573 (22)	384 (12)	157 (−)	1995 (46)	270 (27)	322 (0)	n/a
CRC surgeries/y	110	250	120	–	200	–	86	340	80	–	n/a
FTE colorectal surgeons	3	4	3	5	3	1	2	3	2	–	
Teaching status	N	Y	Y	N	Y	Y	Y	Y	N	N	n/a
Patients pre post	20 20	20 20	20 17	20 20	10 20	20 20	20 20	20 20	20 20	20 14	190 191
Interviewees	CNS^a^ CS N	CS N D QO	CS (2) HN (2)	CS D^b^ QO^b^	CNS HN QO	CS G / I HN N^a^ Q^a^	CS N QO	CS HN QO	G / I	CS	30

^a^Telephone interview

^b^Also provided information on hospital I and J

*CNS* Clinical nurse specialist, *CS* colorectal surgeon, *D* Dietician, *G / I* Gastroenterologist / internist, *HN* Head nurse, *N* Nurse, *QO* Quality officer

Our findings revealed an overall improvement in AR of 6%, varying from − 13 to + 22%, and a statistically significant average increase from 56 to 62% (*p* < 0.00001). No significant change in AR was observed at 3 of the hospitals. Findings from one hospital revealed a reduced AR in the post-test; however, six of the ten hospitals exhibited significantly higher ARs. Overall, LOS decreased significantly by 2.1 days (*p* = 0.0230). However, there was considerable variation, ranging from a decrease of 5.06 days to an increase of 2.15 days [18].

Table 3 includes results from the quantitative evaluation of the primary and secondary outcomes. Based on pre-test median adherence we compared results from the 5 top-scoring hospitals with those from the 5 lowest-scoring hospitals to test the hypothesis that those with lower pre-test scores would exhibit higher IRs. The mean IRs for the top 5 vs. the bottom 5 hospitals were 0.2% (range of − 13 to 12%) vs. 11.2% (range of − 1 to 22%), respectively, although this difference did not reach statistical significance (*p* = 0.17384).

**Table 3:** Protocol adherence, improvement rate, LOS and ΔLOS

H	Pre-test median adherence	Post-test median adherence (ranking)	Improvement rate (ranking)	Post-test mean LOS in days (ranking)	Δ mean LOS in days (ranking)	Overall ranking	Self-rated adherence
1	65%^a^	75% (1)	10% (4)	6.0 (1)	−3.1 (4)	10 (1)	88%
2	43%^b^	65% (4)	22% (1)	8.2 (2)	−4.2 (3)	10 (1)	60%
3	51%^b^	67% (3)	16% (2)	8.5 (3)	−2.0 (6)	14 (3)	71%
4	56%^a^	68% (2)	12% (3)	9.9 (5)	−2.4 (5)	15 (4)	88%
5	52%^b^	51% (9)	−1% (7)	10.2 (6)	−5.0 (1)	23 (5)	59%
6	46%^b^	55% (7)	9% (6)	9.5 (4)	−1.7 (7)	24 (6)	–
7	54%^b^	64% (5)	10% (4)	17.0 (9)	1.7 (8)	26 (7)	79%
8	60%^a^	47% (10)	−13% (10)	10.3 (8)	− 4.4 (2)	30 (8)	71%
9	57%^a^	54% (8)	− 3% (8)	10.2 (6)	2.1 (10)	32 (9)	72%
10	69%^a^	64% (5)	−5% (9)	18.8 (10)	1.8 (9)	33 (10)	64%

^a^Top-5 hospitals pre-test median adherence

^b^Bottom-5 hospitals pre-test median adherence

**Fig. 2:**
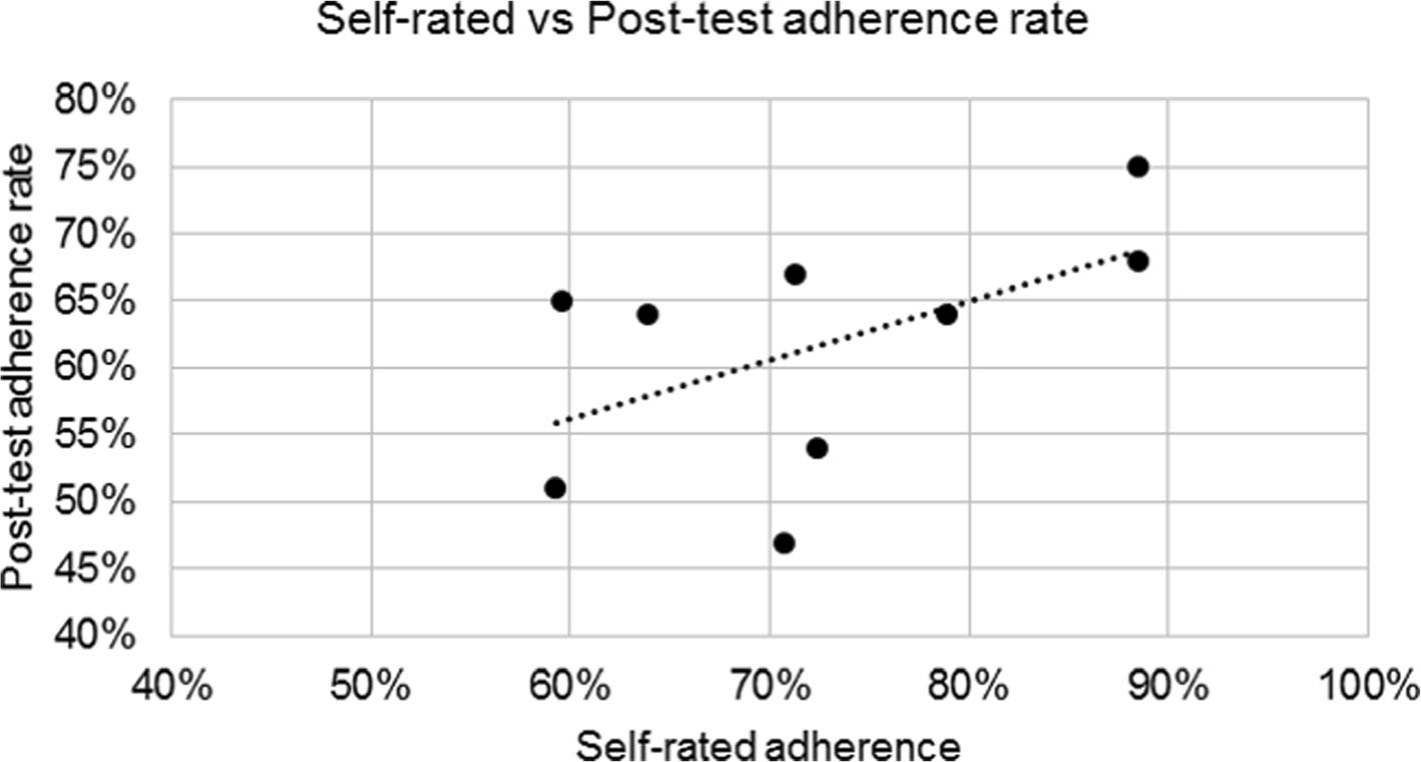
Self-rated adherence (SrA) vs. post-test median adherence rate (AR)

The correlation between intended adherence (self-rated) and measured adherence (median adherence post-test) is shown in Fig. 2. Our findings revealed a small positive correlation between SrA and median adherence that did not reach statistical significance (Pearson’s R = 0.5358, R^2^ = 0.2871, *p* = 0.13706).

### Case studies

As shown in Table 3, Hospitals 1 and 2, each with scores of 10 points, were included as high-performing cases (i.e., hospitals that scored the highest in the overall rankings as described). Hospitals 8, 9, and 10 were included as low-performing cases (i.e., hospitals that scored lowest in the overall rankings). For each case, we prepared a short description that reviews the main quantitative findings and the experiences of professionals involved with a focus on the four core constructs of eNPT (Additional file 1).

Joint display are presented that include data from the highest-ranking (Hospitals 1 and 2) and lowest-ranking (Hospitals 8, 9, and 10) cases, followed by a short explanatory text that includes illustrative quotes that facilitate further comparison.

***Capability***, the first construct of eNPT, includes the possibilities offered by the complex intervention in terms of workability and integration into a social system. As shown in Table 4, the workability of the CP was perceived as positive in four of the cases. The CP was perceived as having a minimal impact on workload and served to increase both structure and patient safety.

*“The doctors worked the model pathway in our treatment standards.” (Hospital 2).*

*“At the start, yes, in the beginning. Now maybe we profit. But at the start we had to explain and tell everyone …*. *Now, it is … when it works, it works. When the patient arrives and everything is clear, it is a positive effect.” (Hospital 10).*

**Table 4:** Joint display capability

Capability: Possibilities presented by the complex intervention (Workability & Integration)					
	AR (IR)	SrA	↑↓	LOS (ΔLOS)	Qualitative data
**Hospital 1**	75% (10%)	88%	↑17 ↓5	(6.0d) (−3.1d)	• CP implemented before project, project used to update and adapt • CP integrated in electronic patient record • No effect on workload • Standardization, monitoring mentioned as standard ways of working
**Hospital 2**	65% (22%)	60%	↑18 ↓3	8.2d (− 4.2)	• CP implemented during project • CP not integrated in patient record, but integrated in work processes • Initial increase in workload • Delicate process to reach consensus
**Hospital 8**	47% (− 13%)	71%	↑6 ↓9	10.3d (− 4.4)	• No CP implemented • Local protocol not integrated in the patient record • Using protocol decreases workload • Perioperative care is unstructured, depending on individual preferences
**Hospital 9**	54% (−3%)	72%	↑13 ↓6	10.2d (2.1d)	• CP partly implemented during project, not integrated in patient record • No effect on workload • Ambivalent perception of standardization: clarity versus ‘cook book medicine’ and loss of autonomy
**Hospital 10**	64% (−5%)	64%	↑7 ↓8	18.8d (1.8)	• CP implemented during project • CP integrated in (paper based) patient record • Decrease in workload • Standardization perceived as positive providing clarity and safety

*AR* Adherence rate post-test, *IR* Improvement rate, *SrA* Self-rated adherence, ↑↓ number of interventions on which adherence went up or down, *LOS* length of stay post-test, *∆LOS* change in mean LOS (days)

However, respondents from Hospital 9 expressed doubts regarding feasibility and standardization.

*“So that’s what we decided. Okay, because they were the same, it was dubious to get them up the first day. But, what they do recommend is that they have an evaluation by physio, or … well with them it is a … well an evaluation at least.” (Hospital 9).*

Hospital 1 and Hospital 10 integrated the CP within the existing patient record. In Hospital 2, although the CP was not integrated into the patient record, it was integrated into the work processes. However, we note that the respondents perceived the process of reaching consensus as somewhat difficult. By contrast, perioperative care provided by Hospital 8 was characterized as unstructured and the CP was not implemented. In Hospital 9, part of the CP was implemented, but it was not integrated into the overall program.

In summary, implementation of the CP and associated improvements in performance were facilitated by the overall workability and practical nature of the CP, its clarity and safety, and its integration into pre-existing work processes.

***Capacity*,** the second construct of eNPT, is defined as the social-structural resources available to agents. Findings presented in Table 5 reveal that resources, including time for multidisciplinary team meetings and a data system, were available only in Hospital 1. All other hospitals reported both resource and time constraints. Most notably, the lack of an automated data system for monitoring performance served as a barrier to implementation of the CP. Interestingly, we note that the hospital with the highest IR also reported limitations with respect to resources. Furthermore, in all hospitals except for Hospital 9, teamwork and collaboration were perceived as strong.

*“And in fact we have no departments-life. We are not meeting together, except in the corridor and so on, but we have no regular meeting for routine problems or so.” (Hospital 9).*

**Table 5:** Joint display capacity

Capacity: Social-structural resources available to agents (Social roles, Social norms, Material & Cognitive resources)					
	AR (IR)	SrA	↑↓	LOS (ΔLOS)	Qualitative data
**Hospital 1**	75% (10%)	88%	↑17 ↓5	(6.0d) (−3.1d)	• Resources available, including time and data system • No support from quality department, but trained CP facilitator supported project • Clear clinical leader • Improvement team had no experience in CP methodology, project as opportunity to learn each other’s contribution
**Hospital 2**	65% (22%)	60%	↑18 ↓3	8.2d (−4.2d)	• Resources and time constraints. Comprehensive data system available, but manual retrieval of data • Improvement team had experience with developing and implementing CPs, a detailed project plan was used, quality department supported the project • Medical champion present, but new in hospital, perceived as disadvantage in collaboration with surgeons
**Hospital 8**	47% (−13%)	71%	↑6 ↓9	10.3d (−4.4d)	• No resources and no time, no data system available • No clear local champion • Day-to-day teamwork perceived as good
**Hospital 9**	54% (−3%)	72%	↑13 ↓6	10.2d (2.1d)	• No resources nor time for improvement activities, no data system available. • No improvement team formed, and no clear clinical leader • Limited support from quality department • Day-to-day teamwork perceived as challenging
**Hospital 10**	64% (−5%)	64%	↑7 ↓8	18.8d (1.8d)	• Lack of resources and time, staff shortage, limited data available in data system • Both medical and nursing champions, but medical champion only working on 1 of 2 wards • Improvement team had no experience in CP methodology, champion had experience

*AR* Adherence rate post-test, *IR* Improvement rate, *SrA* Self-rated adherence, ↑↓ number of interventions on which adherence went up or down, *LOS* length of stay post-test, *∆LOS* change in mean LOS (days)

The improvement team in Hospital 1 had no previous experience with the implementation of CPs but received support from trained CP facilitators. The team in Hospital 2 had experience with CP methodology and was supported by the quality management department. In Hospital 10, only the individual who was promoting the program (i.e., the “local champion”) had experience in CP methodology, and in Hospitals 8 and 9, no improvement team was formed. These observed differences suggest that experience with CP methodology and critical support correlate with performance.

The role of the individual responsible for promoting the program, otherwise known as the “local champion” was different in all of the hospitals evaluated. In Hospitals 1 and 10, there were clear local champions of the CP program among the medical and nursing staff (albeit on only one of two participating wards in Hospital 10). In Hospital 2 there was one local champion on the medical staff, but this individual was new to the hospital. No individuals were championing this program in Hospitals 8 and 9.

Thus, we conclude that successful implementation of the CP was hindered by the lack of an automated data system that could be used for feedback purposes but was facilitated by experienced improvement teams or teams that received support for implementing CP methodology. Interestingly, the lack of resources presented no barrier in Hospital 1 as opposed to what was reported by the other hospitals. The roles played by teamwork and the local champion remain ambiguous.

***Potential***, the third construct in eNPT, includes individual intentions and the collective commitment of all agents. Findings presented in Table 6 reveal that willingness to change was perceived as intrinsic among the staff at Hospitals 1, 2, and 10, and that feedback on the pre-test performance acted to trigger efforts toward improvement. At Hospital 8, individual ways of working were reported. However, the most striking differences between the hospitals that exhibited high vs. low improvement included the relative status of the personnel involved in the decision to join the project and specific CP strategy. For example, in Hospitals 8, 9, and 10, the decision to join the project was made by middle management or by the improvement team itself. By contrast, in Hospitals 1 and 2, this decision was made by higher-level management. Similarly, in Hospitals 1 and 2, CP development was an integral part of the hospital strategy, while this is not the case in any of the other hospitals. There was a remarkable contrast regarding the nature of “normal” quality improvement strategies when comparing Hospitals 1 and 2 to Hospitals 8, 9, and 10. Respondents from Hospitals 8 and 10 reported deep differences in the approaches taken by management and clinicians.

“*… always on the conflict between an administrative approach and a medical approach, huh. So it’s that gap and it’s been going on for years” (Hospital 8).*

**Table 6:** Joint display potential

Potential: Social-cognitive resources available to agents (Individual intentions & Collective commitment)					
	AR (IR)	SrA	↑↓	LOS (ΔLOS)	Qualitative data
**Hospital 1**	75% (10%)	88%	↑17 ↓5	(6.0d) (−3.1d)	• Willingness to change was present, team wanted to improve further • Quality improvement is considered important within hospital • CP development is team effort, with collective goals • CP development aligned with hospital strategy, higher management decided to join the project
**Hospital 2**	65% (22%)	60%	↑18 ↓3	8.2d (− 4.2d)	• Improvement team was motivated • Motivation hampered by conflicting priorities • Identifiable collective reason to start project • CP development aligned with hospital strategy, higher management decided to join the project
**Hospital 8**	47% (−13%)	71%	↑6 ↓9	10.3d (−4.4d)	• Little motivation and collective commitment • Certification, external pressure as leverage for CP development • Conflict of views on quality: administrative vs clinical approach • CP development not aligned with hospital strategy, middle management decided to join the project
**Hospital 9**	54% (−3%)	72%	↑13 ↓6	10.2d (2.1d)	• Lacking shared goals and commitment • External pressure provides leverage for CP development • Management not involved, quality improvement as ‘part of the job’ • CP development not aligned with hospital strategy, team decided to join the project
**Hospital 10**	64% (−5%)	64%	↑7 ↓8	18.8d (1.8d)	• Feedback of the pre-test data acted as trigger, team intrinsically motivated • Quality improvement perceived as important part of the job, project as opportunity to update local protocols, benchmark and learn • CP development is a team effort, with shared ambitions, but more so on the ward where medical champion worked • Little to no support by management, and different views on quality between management and clinicians • CP development is not aligned with hospital strategy, middle management decided to join the project

*AR* Adherence rate post-test, *IR* Improvement rate, *SrA* Self-rated adherence, ↑↓ number of interventions on which adherence went up or down, *LOS* length of stay post-test, *∆LOS* change in mean LOS (days)

Hospitals 8 and 9 reported that external pressure worked to facilitate standardization of care. This was not mentioned in reports from any of the other hospitals. Finally, it was observed that the teams in Hospitals 1 and 2 have clear objectives and priorities.

*“And that fine-tuning … we first looked to see where there is room for improvement. So we set a number of general goals, of which the most remarkable was, say, reducing the admission, the length of stay, but also reducing nausea. In our analysis, these sprang out.” (Hospital 1).*

In short, implementation of the CP was facilitated by the team’s intrinsic motivation to work on specific goals and priorities and by the fact that CP development is part of the hospital’s overall strategy. Individualism, external pressure, perceptions of serious differences between the managerial and clinical approaches to patient care, and decisions to join the project made by the middle, as opposed to the upper-level management, were all barriers to implementation.

***Contribution***, the fourth and final construct of eNPT, refers to the role of various agents in the process of implementation of a complex intervention. This would include providing explanations, cognitive participation, actions, and reflexive monitoring. As shown in Table 7, the intervention was seen as “making sense” in all cases. The model CP was practical and clear and was valued for its evidence base. Feedback as part of the intervention was also seen as important. Positive outcomes were expected at Hospitals 1 and 10, while the expectations were more ambivalent at Hospital 2. The improvement teams were critical to the content of the CP at Hospitals 1, 2, and 10. Interventions were scrutinized and in some cases adapted before implementation.

*“Yes, I have seen that. Except … we already had everything [laughs]. So yes, it did not contain much news for us.” (Hospital 1).*

**Table 7:** Joint display contribution

Contribution: What agents do to implement a complex intervention (Coherence, Cognitive participation, Collective action & Reflexive monitoring)					
	AR (IR)	SrA	↑↓	LOS (ΔLOS)	Qualitative data
**Hospital 1**	75% (10%)	88%	↑17 ↓5	(6.0d) (−3.1d)	• Model CP as refresher, evidence base valued, feedback shared in improvement team • Positive expectation of patient and team outcomes • Benchmarking with other hospitals valued • 9 disciplines involved • Activities: updating protocol, training, communication, meetings • Feedback and monitoring perceived as crucial, and routinely used • Plans ready for future development of CP
**Hospital 2**	65% (22%)	60%	↑18 ↓3	8.2d (−4.2d)	• Evidence base of model CP valued, feedback from pre-test discussed with individuals • Ambivalent outcome expectations • Benchmarking with other hospitals valued • 5 disciplines involved • Activities: updating protocol, meetings, mandatory training, laminated poster, development of CP took longer than expected • Follow-up of data, monitoring and feedback perceived as frustrating due to manual data retrieving • Plan for further development
**Hospital 8**	47% (−13%)	71%	↑6 ↓9	10.3d (−4.4d)	• Model pathway perceived as logical, clear (but not implemented) • CP could help to organize some of the care, positive • Unclear if and how feedback from pre-test was communicated • No improvement team, no activities
**Hospital 9**	54% (−3%)	72%	↑13 ↓6	10.2d (2.1d)	• CP desired, but unknown, questioning applicability of some interventions, unclear if feedback was spread • No change in patient outcomes was expected • Benchmarking with other hospitals valued • 4 disciplines involved • Activities: updating protocol, limited training, crucial role for head nurses • Feedback and monitoring perceived as crucial, but not used routinely • Desire to develop more CPs and work with improvement team
**Hospital 10**	64% (−5%)	64%	↑7 ↓8	18.8d (1.8d)	• Model CP valued, questioning applicability of some interventions, feedback shared beyond improvement team • Positive expectation of patient and team outcomes • Benchmarking with other hospitals valued • 4 disciplines involved • Activities: updating protocols, meetings, 1-on-1 instructions, communication, CP printed in patient record (reminder) • Feedback and monitoring is used, a number of indicators from the model CP was added for routine monitoring • Plan for new patient record analysis

*AR* Adherence rate post-test, *IR* Improvement rate, *SrA* Self-rated adherence, ↑↓ number of interventions on which adherence went up or down, *LOS* length of stay post-test, *∆LOS* change in mean LOS (days)

The number of disciplines involved in the implementation process was considerably larger in Hospitals 1 and 2; by contrast, in Hospitals 8 and 9, the absence of physicians was noticeable. All cases except Hospital 8 described a variety of implementation activities, including training and updating the local protocol.

*“And so the care pathway is explained step-by-step, with the intention to receive comments.” (Hospital 2).*

*“The care pathway is in the patient record, it is printed for the colleagues, and also available in intranet. And I try to make sure everybody knows that.” (Hospital 10).*

Another noticeable difference was that team-training was not organized in Hospital 10. Reflexive monitoring and the use of feedback to improve performance was regarded as important at all hospitals. It was remarkable that one of the higher-performing hospitals reported the greatest struggles in collecting feedback data. International benchmarking with other hospitals was also valued in all cases, although it was not clear how the feedback was shared or how benchmarking was perceived at Hospitals 8 and 9.

*“And to be able to compare ourselves to other hospitals, which we have never ever done before, you know we rarely have some benchmarking.” (Hospital 9).*

*“I thought that was a good thing, that really was thought-provoking. One should compare oneself with other hospitals.” (Hospital 10).*

All teams that implemented the CP indicated they have ideas and plans for future development and suggested that the implementation might “carry...forward in time and space” [22].

In summary, implementation of the CP and high performance was facilitated by the fact the intervention made sense to the healthcare staff. However, positive expectations were not sufficient to achieve positive outcomes. Additional facilitators might include the use of international feedback data and involvement of all relevant disciplines, as the absence of physician involvement was observed to be a barrier to improved performance.

## Discussion

### Main results

The primary outcomes of this study included median protocol adherence and IR. Protocol adherence improved overall. Among the 10 hospitals ranked in order of pre-test adherence, we observed a difference in mean IRs of 0.2 and 11.2% for the 5 highest-ranked and the 5 lowest-ranked hospitals, respectively, although this difference did not achieve statistical significance. The lack of statistical significance might be attributed to the small sample size and/or to the wide variation in IRs.

The secondary outcomes of our study were mean LOS and SrA. While mean LOS decreased by 2.1 days, a decrease in LOS was not observed in all participating hospitals. This is in contrast to the results presented by Larson et al. [25] that focused on the collaborative implementation of a colorectal cancer CP in which reductions in LOS were achieved by all participating teams. A possible explanation for this discrepancy could be that the focus of our study was protocol adherence, as opposed to LOS.

We were unable to establish a relationship between SrA and post-test AR. We did observe, however, that 7 of the 9 hospitals in our study *overestimated* their performance. A systematic review by Adams (1999) of self-reporting bias in guideline adherence revealed an absolute overestimation of 27% [26]. The difference between self-reported and measured adherence in our study was less than 27%, although it is clear that the overestimation of SrA remains a problem.

We also observed differences in improvements in both protocol adherence and LOS. LOS is used as a primary outcome measure in most studies focused on ERAS or fast-track protocols. Recently, Balvardi et al. (2018) suggested LOS could be used as a measure of in-hospital recovery with equal construct-validity as “readiness for discharge.” [27]. However, due to the small number of patients per hospital in this study (≤20), LOS (and ∆LOS) should be interpreted with caution. This is among the reasons underlying our decision to use both LOS and protocol adherence for ranking and hospital selection.

### Implementation process

Implementation of the CP differed between the hospitals. While there were minor differences in *capability*, as the workability of the CP was perceived as positive by all, its integration into work processes was stronger in hospitals with higher IRs. Hospital 1 had already implemented a CP before the start of our project; as such, our project was used as a means to update the local CP. Nonetheless, Hospital 1 improved its AR by 10%. These results suggest that, although they already had a CP in place, they may have used it more effectively as a result of participating in our project. In Hospital 2, a CP was developed from the start, although some of the care recommended by the ERP was already provided (i.e., 43% pre-implementation adherence). As proposed by eNPT, the capability of working successfully with a complex intervention depends on both its workability and its integration [22]. Adapting the ERP to fit local circumstances has been identified as a key facilitator of implementation [12]. Furthermore, the importance of integrating new ways of working within given systems was previously described in a study that featured an earlier iteration of eNPT in colorectal surgery [8]. This information provides some explanation for observed differences between high- and low-performing hospitals.

For *capacity*, there were more noticeable differences between the hospitals that could explain differences in IRs. The level of experience and support for using CP methodology provided to the improvement team appears to be directly related to the IR. This result is consistent with findings reported in previously published studies for colorectal surgery [1, 8, 11, 25, 28] as well as in other settings [29, 30]. High IRs were achieved in hospitals in which a trained facilitator or quality management officer provided support for the improvement team. A lack of resources is a well-documented barrier to implementation [10–12, 31–34]. However, our data suggest both high- and low-performing hospitals experienced a lack of resources. Three of the 5 hospitals had a “local champion” who provided support for the initiative. Hospital 1 identified a clear and institutionally-sanctioned champion. By contrast, the champion in Hospital 2 was relatively new to the hospital; this was perceived as disadvantageous. In Hospital 10, the champion worked on only one of two wards participating in this initiative, and respondents indicated that implementation of the CP was less successful on the second of the two wards. Coxon et al. (2017) developed a program theory based on the concept of “change agency” in which the change agent is identified as a clinical local champion. The authors suggest that local champions of these initiatives should have strong clinical skills and know-how, need to be familiar with the local situation, and should have good management and people skills [28]. One proposition of the eNPT states that the incorporation of a complex intervention in a given social system depends on the users’ capacity to cooperate and coordinate their actions [22]. Teams in the high-performing hospitals had superior access to cognitive resources (experience, training, facilitation) which facilitated cooperation and coordination of their actions. However, the roles of the local champion, teamwork and material resources in our hospital cases remain ambiguous.

The first observable difference between high- and low-ranking hospitals in *potential* was that intrinsic motivation, shared goals, and overall commitment were reported in Hospitals 1 and 2, but were lacking at Hospitals 8 and 9. In Hospital 10, the team was motivated, although the team on the ward where the medical lead, or local champion, worked showed more commitment than did the team on the other ward. This could be one explanation for low IRs found at this hospital. Previous research supports the importance of staff morale and commitment when implementing CPs. For example, Jabbour et al. (2018) identified strong commitment as a facilitator for the implementation of CPs in a complex environment [35]. Other studies focusing on the implementation of ERAS also identified commitment as facilitator [10–12, 28]. Lack of commitment observed in Hospitals 8 and 9 could explain their low performance. Second, the view of CP development as part of the overall hospital strategy (Hospitals 1 and 2) and the perceived differences with respect to quality improvement reported among clinicians and managers (Hospitals 8, 9, and 10) could contribute to the observed differences in performance. In eNPT, individual intentions and shared commitment are concepts that are used to operationalize potential. This theory proposes that translation of capacity into collective action depends on participants’ potential (and thus intentions and commitment) for the successful enactment of complex interventions [22]. Numerous papers have described the importance of management support for quality improvement, including the systematic review by Kringos et al. (2015), as well as individual studies that have examined the implementation of CP or ERAS protocols [8, 11, 12, 25, 31, 36–38]. These studies suggest that management endorsement and support are key factors that promote success. Lack of management support in Hospitals 8, 9, and 10, including the management level at which the decision to join the project was made, together with a CP development program that was not aligned with hospital policy could explain the low performance.

A focus on the final core construct, *contribution*, revealed several interesting results. In all cases, the intervention was valued and made sense to the users, although in Hospital 1, implementation of the model CP and feedback were perceived as a routine practice. Coherence or sense-making in eNPT terms involves the assignment of meaning to a specific intervention [22]. This can be seen as the first important step towards normalization of a given intervention, as has been described in previous research. Banks et al. (2017) note that a “clear understanding and acceptance of the aims of the project, including the legitimacy of the research data and the process of pathway development” (p.109) can lead to agreement and implementation [39]. Both high- and low-ranking hospitals exhibited sense-making and expectations of positive outcomes. As such, our findings suggest that these attributes are not sufficient to achieve positive outcomes.

We observed no meaningful distinctions between the hospitals regarding implementation activities used, except for Hospital 8, where there were no implementation activities. In our previous research, we identified implementation activities focused on competence, behavior, or workplace [15]. We noticed that, in all cases, implementation involved activities from all three categories. However, we did observe differences with respect to the involvement of relevant disciplines. There was a noticeable absence of physician involvement at the low-performing hospitals (Hospitals 8 and 9). This relates to the concept of cognitive participation as defined by eNPT and the level to which users choose to participate in a complex intervention and become members of a community of practice [22]. The importance of building a community of practice was also discussed by Gotlib Conn et al. (2015), who identified this as a key component of successful implementation [8]. This was also considered in the study of Larson et al. [25] that focused on the collaborative implementation of a CP for colorectal cancer surgery.

All hospitals save for Hospital 8 used feedback as important implementation activity. A systematic review revealed that audit and feedback play a role inpromoting effective changes to current practice [40]. Audit and feedback were also identified as key facilitators of the implementation of an ERP [12]. Audit and feedback, also known as reflexive monitoring in eNPT terminology, are important for the reconfiguration of actions and social relations that are necessary to normalize a given intervention [22]. We observed no differences in the perceived importance and use of feedback that could explain the differences in performance among the hospitals in our study.

**Fig. 3:**
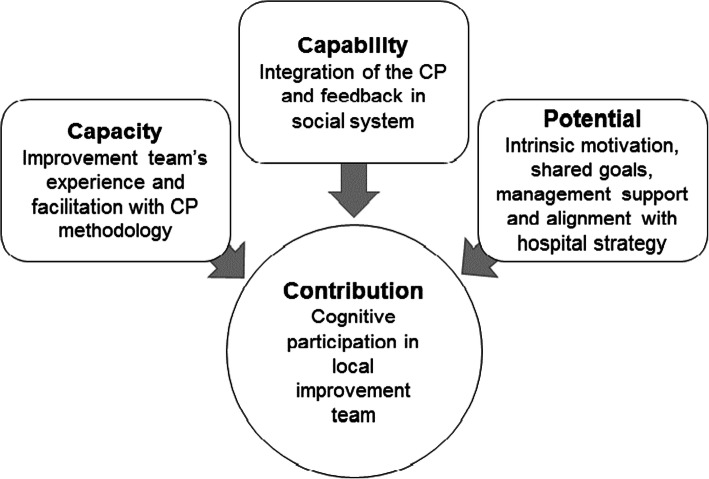
Factors contributing to the differences observed in pre- and post-implementation of the CP (adapted from May, 2013)

As proposed in eNPT, the core constructs capability, capacity, and potential, all have an impact on contribution, which are the actions taken that serve to implement the intervention. In the end, the implementation and normalization of a complex intervention depend on continuous contributions from all users [22]. Fig. 3 highlights factors that may explain the differences between pre- and post-implementation performance. This figure is based on the “resources and possibilities for agents’ contributions to implementation processes” as described by May et al. and links the four main constructs [22]. The findings provide a specific focus on the factors that were present in high-performing hospitals and that were absent among those that were low-performing. Other factors, including workability of the CP, availability of resources, sense-making, collective and diverse implementation activities, and the use of reflexive monitoring, have been reported as important factors in the implementation process and were present in both the high- and low-ranking hospitals.

Factors present in high-performing, but not in low performing hospitals.

### Strengths and limitations

The study was performed over a period of 2 years. During this time, the participating teams had the opportunity to review their processes and to develop, improve, implement, and normalize their CPs. A major methodological strength of this study is that the interviews were performed and initially coded before the quantitative data were analyzed. This strategy serves to reduce interpretation bias [21]. The selection of hospitals from both the high and low ends of the performance spectrum ensured that information-rich cases would be included.

Our study has several limitations. Because it was not feasible to include all 10 hospitals in the analysis, we selected the top 2 and bottom 3 hospitals based on the ranking data presented in Table 3. This was an arbitrary selection, and we recognize that other selection strategies were possible. The ranking shows that the hospitals ranked 1–4 exhibit total ranking scores between 10 and 15 points, while the hospitals ranked 5–7 have total scores between 23 and 26 points, and the hospitals ranked 8–10 scored between 30 to 33 points. These findings suggest that the 10 hospitals can be divided into high, intermediate, and low performers. To validate our selection of the top 2 and bottom 3 hospitals, we compared some of our findings with those from the intermediate group (Hospitals 3–7). The characteristics of Hospitals 3 and 4 were similar to those described in detail for Hospitals 1 and 2. This suggests that including data from Hospitals 3 and 4 would provide no additional insights and that we captured ample data on high performance in our analysis that included only Hospitals 1 and 2. The hospitals in the intermediate group showed a more diverse picture. Some characteristics were similar to those of the high-performing hospitals (e.g., motivation, the involvement of a local champion, and variety of implementation activities) while some characteristics were similar to those of the low-performing hospitals (e.g., few resources and lack of support from management, as well as low levels of collective commitment and support/training in CP methodology). These findings were anticipated and stand in support of our decision to limit our analysis to findings from the top and bottom hospitals based on our ranking profile.

Hospitals 8 and 9 had a joint quality management officer and project support. As such, it was not always clear how to assign applicable responses. Furthermore, interviews were conducted with only 3 or 4 professionals who were directly involved at each hospital and, as such, we may have only a limited account of the implementation process. To mitigate this, we used data triangulation methods and checked interview data with field and project notes, which is an established method to enhance the trustworthiness of data [41].

Given the importance of improving protocol adherence and reducing LOS, additional research is warranted to increase our understanding of the contributing factors identified in our study (Fig. 3). Further research might focus on the effort to achieve data saturation at a single hospital, as opposed to data saturation for the overall sample. Similarly, audits in those hospitals using the CP or a longitudinal quantitative study might help to determine whether the CP was normalized in one or more of these cases.

## Conclusions

Our study combined quantitative and qualitative data and revealed that a change in protocol adherence does not automatically lead to a change in LOS. Overall improvement in both protocol adherence and LOS was achieved, although the findings were highly variable among the hospitals studied.

Multiple factors in the implementation process could contribute to the differences in the IRs observed here. Conceptualization of these factors using eNPT suggests that teams that can integrate the CP into their social system, those that have experience or that receive support for the implementation of CP methodology, as well as those that are intrinsically motivated, capable of working towards shared goals, receive active management support, and are employed in environments in which CP development is aligned with the hospital strategy are ultimately more successful at the implementation of a CP for colorectal cancer surgery.

Our conclusion implies that multidisciplinary teams intending to implement a CP should invest in shared goals and teamwork and should focus on the integration of the CP into daily processes. Support from hospital management directed specifically at quality improvement may likewise facilitate the implementation process.

## Supplementary Information

**Additional file 1.** Case descriptions

## Data Availability

The datasets generated and/or analyzed during the current study are not publicly available due them containing information that could compromise research participant privacy/consent, but are available from the corresponding author (RvZ) on reasonable request.

## Abbreviations

AR: Adherence Rate
CP: Care Pathway
DSM: Diagnostic and Statistical Manual of Mental Disorders
ERAS: Enhanced Recovery After Surgery
ERP: Enhanced Recovery Protocol
IR: Improvement Rate
LOS: Length Of Stay
(e)NPT: (extended) Normalization Process Theory
SrA: Self-rated Adherence
