# Supplementary material for: A mixed methods multiple case study to evaluate the implementation of a care pathway for colorectal cancer surgery using extended normalization process theory
Source: BMC Health Serv Res. 2021 Jan 4;21:11. doi: 10.1186/s12913-020-06011-w (PMC7784254; doi:10.1186/s12913-020-06011-w)
Supplement: Supplementary file 1 — Additional file 1. Case descriptions [file 12913_2020_6011_MOESM1_ESM.docx]

**Addtional file – Case descriptions**

***Hospital 1***

*Quantitative –* Hospital 1 had a post-test median adherence rate of 75%, with an improvement rate of 10%. The number of interventions on which adherence improved, was 17, of which seven scored an adherence <70% in the pre-test (priority interventions). Adherence decreased in five interventions. Mean LOS in the post-test was 6.0 days, a decrease of 3.1 days.

*Capability* – This hospital already had a CP in place and used the project to adapt this. Respondents did not experience an effect on workload after adaptation. The CP is integrated in the electronic patient record. Also, respondents noted that when required (e.g. due to complications), deviating from the CP was possible. Standardization, measuring and discussing results were mentioned as standard ways of working.

*Capacity* – Both material (time) and cognitive (training) resources were available, including a data system. There was no support from the quality department, but a trained CP facilitator supported the project, together with a formally appointed clinical leader, with good collaboration in the improvement team. The improvement team members knew each other before the start of the project, but had no experience in CP development and implementation. The project offered the possibility to learn each other’s roles in the care process, and look at problems from different perspectives.

*Potential* – Interviewees indicated that willingness to change was present, and that quality improvement is considered important within the hospital. Despite the already good results, the team wanted to improve further. There was a high level of involvement of all disciplines during the project. A number of collective goals (e.g. reduce LOS, improve postoperative nausea) were set. CP development and implementation is perceived and organized as team effort. Development of CP’s is anchored in hospital strategy, and the higher management decided to join the project.

*Contribution –* The model CP delivered to the hospital was perceived as ‘not much news’, was used as a ‘refresher’, although the evidence base of the CP was valued. The project was expected to deliver positive results, and interviewees indicated that teamwork had already improved because of the project. The benchmarking with other hospitals, as part of the intervention, was seen as inspiring, the feedback report was shared in the improvement team. Involved disciplines were: colorectal surgeon, gastroenterologist, nursing, clinical nurse specialist, anesthetist, dietician, patients, physiotherapist, CP facilitator. A wide range of implementation activities was used, including updating protocols, meetings, mandatory training, 1-on-1 instruction, and several communication modes (e.g. weekly email newsletter, linking pin in each involved discipline). This team indicated that they already monitor their performance every three months. And although the results from the pre-test were perceived as good, this did not make the team lean back. A number of goals and indicators was added to the monitoring. Several actions were mentioned to sustain improvement, e.g. repeated training, training for new employees.

***Hospital 2***

*Quantitative –* Hospital 2 had a post-test median adherence rate of 65%, with an improvement rate of 22%. The number of interventions on which adherence improved, was 18, of which 13 scored an adherence <70% in the pre-test. Adherence decreased in three interventions. Mean LOS in the post-test was 8.2 days, a decrease of 4.2 days.

*Capability* – The CP was seen as practical, although with an initial increase in workload. The CP was not integrated in the patient record, but was considered ‘nothing special’ and integrated in the work processes. Developing the CP proved to be a delicate process to reach consensus, because of variation between the surgeons in the hospital.

*Capacity* – Interviewees indicated there were both resource and time constraints. The project was initially supported by a quality officer, but she was not replaced during leave. On the other hand, the improvement team had experience with developing and implementing CP’s. The team used a detailed project plan to guide the project. A comprehensive data system was available in the hospital, but data had to be retrieved manually. The medical champion was relatively new in the hospital, impeding collaboration and teamwork with other surgeons, who do not all follow the CP.

*Potential* – CP development is part of the hospital policy, the chief medical officer supports the project. It was a higher management decision to join the project. The inter-doctor variation reported by the respondents, acted as a collective reason to join the project. The improvement team was motivated to develop and implement the CP, despite a struggle for priorities.

*Contribution* – The evidence base of the CP was valued, interviewees indicated it was needed to convince colleagues. The interviewees were ambivalent in the expected outcomes. Deviations from the pathway were deemed necessary, and were experienced as frustrating when caused by organizational problems. The opportunity to compare with and learn from other hospitals was sees as positive. The feedback was presented and used to introduce the new CP. The following disciplines were involved in the project: colorectal surgeon, nursing, dietician, anesthetist, quality officer. The improvement team used the following activities: updating the local protocol, meetings, mandatory training and a used a laminated poster as reminder for the team. Follow-up of data was perceived as time consuming and difficult because of the manual data retrieving. This was perceived as frustrating and demotivating, making monitoring almost impossible. Development and implementation of the CP was perceived as time consuming, taking ‘longer than expected’. The interviewees expected that a newly developed patient brochure will further help, not only to better inform patients, but also to sustain the CP.

***Hospital 8***

*Quantitative –* Hospital 8 had a post-test median adherence rate of 47%, with an improvement rate of -13%. Despite the overall decrease in adherence, the number of interventions on which adherence improved, was six, of which five scored an adherence <70% in the pre-test. Adherence decreased in nine interventions. In the post-test, the mean LOS was 10.3 days, a decrease of 4.4 days.

*Capability* – In this hospital, interviewees indicated that the interventions presented in the model CP are used in practice, but in a different format, which is not integrated in the patient record. The use of a protocol, of the standardized interventions, was perceived to decrease workload and save time. However, preoperative care was characterized as an unsystematic process, depending on the individual surgeon.

*Capacity* – Doctors are encouraged to improve quality, but there are no resource and no time. There was no data system available. A clear local champion to promote the CP was not available; a study nurse was perceived as being the project leader. Daily teamwork is perceived as good; there is a well-structured surgical service.

*Potential* – The respondents indicated that individual doctors do as they want. CP development is not part of a central hospital strategy. There is a struggle between administrative quality (‘ticking boxes’) versus clinical quality (focus on patient): ‘we are here because the patient is important’. Middle management decided to join the project. Certification of services is seen as central strategy, which according to interviewees, ‘unites people’. There is pressure from national health authorities which have imposed national indicators to which the hospital has to comply. This provides leverage to create a more standardized service and improve adherence.

*Contribution* – The model CP describes the same items as those used for certification, it has a logical and clear format. Implementing the CP could help to organize some of the care, which ‘would be good’. Interviewees indicated that feedback and monitoring are crucial for quality control. It was unclear if and how the feedback on the pre-test performance was communicated. The feedback report was send to all surgeons and the medical director. However, other interviewees did not recall to have seen the report, and were not aware of a meeting were the feedback was presented.

There was no improvement team in this hospital, and no improvement project or activities were performed. Interviewees agreed that internal communication regarding the project should have been better.

***Hospital 9***

*Quantitative –* Hospital 9 scored a post-test median adherence rate of 54%, with an improvement rate of -3%.The number of interventions on which adherence improved was 13, of which 9 were priority interventions. Adherence decreased in six interventions. In the post-test the mean LOS was 10.2 days, an increase of 2.1 days.

*Capability –* There were doubts about the feasibility of the CP in this hospital. Not all interventions were directly applicable. The CP was not integrated in the patient record, but protocols were updated. The respondents indicated workload had not changed. There were mixed feelings about standardization; this could enhance structure and safety, but there was also a fear of ‘cook book medicine’. Physicians have great autonomy to deviate from standards.

*Capacity –* Lack of resources, time and a data system was discussed by the respondents. Quality improvement work is regarded ‘part of the job’. There was limited support from the quality department, no facilitation. A clear clinical lead was not identified, involvement of physicians was low. Respondents agreed that collaboration and working relations within and across disciplines were not good.

*Potential –* Respondents indicated that the project had not been communicated to the hospital CEO. Management was interested, but did not support the project actively. It was perceived that the team decided to join the project, however, a sense of shared goals and commitment was lacking. CP development is not a part of the hospital strategy, but the new CEO favors it. The national authorities imposed a national indicator for colorectal cancer. The hospital has to show improvement in accreditations. This can provide leverage for CP implementation. On the other hand, accreditations take up time and resources of the quality department and teams.

*Contribution* – The CP was perceived as logic, but the format was different from local forms. Traditional care was perceived as barrier to implement the CP. After the feedback and improvement sessions, there was no follow-up by an improvement team and little improvement in outcomes was expected. There were some implementation activities, mainly updating the preoperative protocol and some training on specific interventions, not on the complete CP. Involved professionals in these activities were surgeons, dieticians, quality officer, and nurses. Respondents indicated that interventions were implemented because there are ‘good head nurses’. This team reported that feedback and feedback sessions are crucial for quality improvement. Providing information and benchmarking was seen as a mechanism to change practice. The feedback on performance, including the international comparison, was received positive. Results were perceived to be good, motivating the team. However, it was unclear if the feedback was spread within the hospital after the feedback sessions. Interviewees reported no structural use of feedback and monitoring. A desire to continue working with an improvement team was expressed during the interviews.

***Hospital 10***

*Quantitative –* Hospital 10 had a post-test median adherence rate of 64%, with an improvement rate of -5%. Still, the number of interventions on which adherence improved, was seven, of which only one scored an adherence <70% in the pre-test. Adherence decreased in eight interventions. Mean LOS in the post-test was 18.8 days, an increase of 1.8 days.

*Capability –*  In this hospital, the perioperative care for colorectal surgery is provided in two separate wards. On both wards, the model CP was perceived as very practical, and expected to reduce workload. The CP was integrated in the patient record. The interviewees indicated that standardization provides clarity and safety, and a basis to evaluate the care process.

*Capacity –* The respondents reported resource and time constraints, and shortage of staff for multiple disciplines, including surgeons and nurses. A discrepancy in vision on priorities between management and staff on the wards was described. There was limited data available in a data system. There were experienced clinical champions (both medical and nursing), but the medial champion is working only in one of the two wards. The interviewees indicated that on the other ward, the implementation was less successful. The collaboration in the improvement team was perceived as good. The medical champion had experience in developing CP’s, the improvement team not. The team received no assistance from the quality department.

*Potential –* The department head favors CP’s, and decided to join the project, which was perceived as opportunity to update local protocols, and to learn from other hospitals. Quality improvement is seen as ‘vital’, were the hospital management considers this part of the job. The feedback after the pretest showed that the performance was close to the top. On other indicators there was room for improvement, which stimulated the intrinsic motivation. CP development is a team effort, with shared ambitions. CP development is not in the hospital policy, but the need for certification and quality of care is.

*Contribution –* Interviewees reported that the model CP was valued. However, the team was critical to some of the interventions in the model CP, questioning the local applicability. Positive outcomes for both patient care and teamwork were expected. The international comparison with other hospitals was valued. Involved disciplines in the project were: colorectal surgeons, nurses, dietician, and physiotherapist. The following activities were used by the improvement team: updating the local protocol, CP as printed reminder in all patient records, meetings, 1-on-1 instructions, and communication during shift handovers. The results of the pre-test were discussed on the wards, and presented to partner-hospitals in the region. Feedback and monitoring is used, and a number of indicators used in the study were added to the routine monitoring. This, and the plan to do another patient record analysis in a years’ time gives the interviewees the confidence that the CP implementation will be sustained.
